# Development and Validation of the Smartphone Addiction Inventory (SPAI)

**DOI:** 10.1371/journal.pone.0098312

**Published:** 2014-06-04

**Authors:** Yu-Hsuan Lin, Li-Ren Chang, Yang-Han Lee, Hsien-Wei Tseng, Terry B. J. Kuo, Sue-Huei Chen

**Affiliations:** 1 Institute of Brain Science, National Yang-Ming University, Taipei, Taiwan; 2 Department of Psychiatry, College of Medicine, National Taiwan University, Taipei, Taiwan; 3 Department of Psychiatry, National Taiwan University Hospital, Taipei, Taiwan; 4 Department of Psychiatry, Far Eastern Polyclinic, Taipei, Taiwan; 5 Department and Graduate School of Electrical Engineering, Tamkang University, Taipei, Taiwan; 6 Department of Computer and Communication Engineering, De-Lin Institution of Technology, Taipei, Taiwan; 7 Sleep Research Center, National Yang-Ming University, Taipei, Taiwan; 8 Department of Psychology, National Taiwan University, Taipei, Taiwan; Research and Development Corporation, United States of America

## Abstract

**Objective:**

The aim of this study was to develop a self-administered scale based on the special features of smartphone. The reliability and validity of the Smartphone Addiction Inventory (SPAI) was demonstrated.

**Methods:**

A total of 283 participants were recruited from Dec. 2012 to Jul. 2013 to complete a set of questionnaires, including a 26-item SPAI modified from the Chinese Internet Addiction Scale and phantom vibration and ringing syndrome questionnaire. There were 260 males and 23 females, with ages 22.9±2.0 years. Exploratory factor analysis, internal-consistency test, test-retest, and correlation analysis were conducted to verify the reliability and validity of the SPAI. Correlations between each subscale and phantom vibration and ringing were also explored.

**Results:**

Exploratory factor analysis yielded four factors: compulsive behavior, functional impairment, withdrawal and tolerance. Test–retest reliabilities (intraclass correlations  = 0.74–0.91) and internal consistency (Cronbach's α = 0.94) were all satisfactory. The four subscales had moderate to high correlations (0.56–0.78), but had no or very low correlation to phantom vibration/ringing syndrome.

**Conclusion:**

This study provides evidence that the SPAI is a valid and reliable, self-administered screening tool to investigate smartphone addiction. Phantom vibration and ringing might be independent entities of smartphone addiction.

## Introduction

The overuse of smartphones has emerged as a significant social issue with growing popularity of the smartphone. “Smartphone addiction” could be considered as one form of technological addictions. Griffiths [1] operationally defines technological addictions as a behavioral addiction that involves human-machine interaction and is non-chemical in nature. A similar behavior pattern, Internet addiction, has been categorized as a type of “substance related and addictive disorder” in Diagnostic and Statistical Manual of Mental Disorders, 5th edition (DSM-5) [2]. It is conceivable the non-substance addictions are conceptualized from the diagnostic criteria for established substance addictions to provide both a bio-psycho-social context and a direction for a comprehensive model of addiction [3], [4]. For example, we have identified five factors, i.e., tolerance, withdrawal, compulsive symptoms, time management, and interpersonal & health problems in Internet addiction [5].

Smartphone serves not only the portable functions of a “phone”, camera, game and multi-media players, but also thousands of mobile application (app) with available Internet. Thus, some symptoms of smartphone addiction might be different from those in Internet addiction. A recent study explored six factors in smartphone addiction [6]. It suggested that smartphone addiction should be conceptualized as a multi-dimensional construct. In that study, however, the range of subjects' age were relatively wide (from 18 to 53 years) and females were predominate [6]. Besides, the definition of the “tolerance” and “withdrawal” in previous study [6] is not identical to those in DSM [2]. Differently, Internet addiction is well-known to be most prevalent in college school students, male gender is one of its important risk factors [7], and commonly coexist with substance misuse [8]. More psychometric testing is warranted to test the construct validity of the instruments for Smartphone addiction.

Phantom vibrations and ringing of mobile phones, an intermittent perception that a mobile phone is perceived as vibrating and ringing when it is not, are prevalent hallucinations in the general population. Our previous longitudinal study demonstrated the two syndromes were associated with stress during medical internship, and severe phantom vibrations and ringing were correlated to anxiety and depression [9]. However, the association between the two novel phenomena of mobile phone, i.e., “phantom vibration/ringing” and “smartphone addiction”, is unknown.

The aim of this study was to develop a self-administered scale based on features of Internet addiction and the smartphone's characteristics, and to identify smartphone addicts. We hypothesized smartphone addiction has many aspects that are similar to those of Internet addiction and substance addiction, such as tolerance, withdrawal, compulsive behavior, and daily-life function disturbance. The Smartphone Addiction Inventory (SPAI) is specifically designed on the basis of the Chen Internet Addiction Scale (CIAS) with its well-organized five-factor structure. This study examined the reliability and verified the construct validity of the new-established Smartphone Addiction Inventory.

## Methods

### Participants

A total of 283 young adults were recruited from the Department of Electrical Engineering and Department Computer and Communication Engineering of two universities in Northern Taiwan during Dec. 2012 to Jul. 2013. The recruitment strategy was based on the potential higher penetration rate of smartphone use among these students. All students with smartphone participated in this study. Of these, 260 were male and 23 were female, with age 22.9±2.0. The study was approved by the Institutional Review Board of National Taiwan University Hospital, which waived the need for written informed consent from the participants, since the data were analyzed anonymously. All clinical investigations were conducted according to the principles expressed in the Declaration of Helsinki.

### Development of SPAI

Two qualified psychiatrists, Lin and Chang, experienced in substance-related disorder and Internet addiction, modified the 26-item Chen Internet Addiction Scale (CIAS) for “smartphone addiction” assessment. The psychometric study of the modified version of CIAS was conducted by Lin with the permission of Chen, in which five subscales were identified by exploratory factor analysis [5]. The term ‘‘Internet’’ was changed to ‘‘smartphone’’. The Mandarin Chinese version of the measure was finalized by an expert panel. The final revisions included the following: (1) Item 4 and 6 were replaced by the semantically similar item 2 and 3 of the 12-item Problematic Cellular Phone Use Questionnaire [10], because the original item could not make sense by simply using “smartphone use” to substitute “Internet use”(2). Due to the uniqueness of smartphone use, item 21, i.e., “viewing smartphone when crossing the street; fumbling with one's smartphone while driving or waiting, and resulted in danger” was added at the end of the scale(3). For item 23, sentence was modified from original “I make it a habit to sleep less so that more time online.” as “I make it a habit to use smartphone and the sleep quality and total sleep time decreased.” (4) For item 25, sentence was modified from original “I fail to eat meals at the usual time because I am using the Internet” The revisions (3) and (4) were according to the characteristic of portability of smartphone distinguished from the “traditional” Internet use via computer. Participants were asked to rate items on a 4-point Likert scale, 1 =  strongly disagree”, 2 = “somewhat disagree”, 3 = “somewhat agree” and 4 = “strongly agree, so that the SPAI total score ranges from 26 to 104.

### Phantom vibration and ringing questionnaire

To avoid biasing respondents, the questionnaire simply stated: “We are asking you to participate in a study about cell phones.” The questions included whether the respondent had experienced phantom vibrations and ringing during the previous three months [9], [11]. For those who reported phantom vibrations or ringing, we also asked how bothersome they were on four-point Likert scale, i.e., 1 = “no phantom vibration/ringing”, 2 = “not bothersome at all” 3 = “a little bothersome”, 4 = “bothersome” or “very bothersome” according to previous dimensional approach study [9].

### Statistical analysis

All statistical tests were carried out using the SPSS version 15.0 for Windows (SPSS, Chicago, IL, USA). Descriptive statistics for the total sample were performed to show the participants' demographic characteristics. The construct validity of the SPAI was examined by the exploratory factor analysis using a principal component factoring estimation method and oblique promax rotation. The scree plot of ordered eigenvalues of a correlation matrix was used to decide the appropriate number of factors extracted. A factor loading of >0.30 was used to determine the items for each factor. Intra-class correlations were calculated for the test-retest reliability, and Cronbach's alpha was calculated for the internal consistency. The Pearson correlations between the subscales (factors) and phantom vibration/ringing were demonstrated.

## Results

### Factor structure of the SPAI

The total scores of SPAI in this study ranged from 26 to 82 (mean: 51.31±11.77). The factor analysis results are shown in Table 1. Four factors with eigenvalues exceeding 1 were extracted, together explaining 57.28% of the whole scale. The overall sampling adequacy of the 26-item scale was tested using Kaiser-Meyer-Olkin, and a high value of 0.93 was reported. The *p*-value of the Bartlett test was less than 0.001, which indicated that factor analysis was appropriate.

**Table 1:** Factor analysis for Smartphone Addiction Inventory (SPAI).

No	Question		Factor loading			
		Mean(SD)	Compulsive behavior	Functional impairment	Withdrawal	Tolerance
7	Although using smartphone has brought negative effects on my interpersonal relationships, the amount of time spent on Internet remains unreduced	1.86(0.69)	.837	−.092	−.114	.049
10	I feel distressed or down once I cease using smartphone for a certain period of time	1.73(0.65)	.600	−.108	.153	.156
20	My life would be joyless hadn't there been smartphone	1.83(0.73)	.570	.121	.082	−.036
18	My recreational activities are reduced due to smartphone use	1.85(0.71)	.520	.337	.055	−.114
6	I use smartphone for a longer period of time and spend more money than I had intended	1.91(0.72)	.518	.138	−.033	.206
22	I try to spend less time on smartphone, but the efforts were in vain	1.86(0.67)	.492	.080	.321	.022
5	I feel very vigorous upon smartphone use regardless of the fatigues experienced	1.92(0.65)	.448	.142	−.012	.229
21	Surfing the smartphone has exercised negative effects on my physical health. For example, viewing smartphone when crossing the street; fumbling with one's smartphone while driving or waiting, and resulted in danger	1.90(0.78)	.435	.272	.166	−.251
11	I fail to control the impulse to use smartphone	1.89(0.68)	.400	.100	.330	.008
13	I feel aches and soreness in the back or eye discomforts due to excessive smartphone use	2.09(0.77)	−.143	.830	−.137	.125
26	I feel tired on daytime due to late-night use of smartphone	1.71(0.69)	.121	.823	−.018	−.229
23	I make it a habit to use smartphone and the sleep quality and total sleep time decreased	1.98(0.78)	.031	.757	.126	−.081
8	I have slept less than four hours due to using smartphone more than once	1.66(0.71)	.182	.704	−.405	.059
15	To use smartphone has exercised certain negative effects on my schoolwork or job performance.	1.89(0.66)	.073	.535	.183	.011
12	I find myself indulged on the smartphone at the cost of hanging out with friends	1.82(0.68)	.464	.466	−.142	.032
17	My interaction with family members is decreased on account of smartphone use	1.78(0.66)	.390	.465	−.047	.083
24	I need to spend an increasing amount of time on smartphone to achieve same satisfaction as before	1.84(0.68)	.231	.353	.177	.213
4	I feel restless and irritable when the smartphone is unavailable	2.46(0.80)	.088	−.247	.810	−.015
2	I feel uneasy once I stop smartphone for a certain period of time	2.18(0.75)	.257	−.345	.756	.137
25	I can not have meal without smartphone use	1.96(0.82)	.281	−.072	.753	−.318
14	The idea of using smartphone comes as the first thought on mind when waking up each morning	2.56(0.89)	−.586	.376	.698	.100
16	I feel missing something after stopping smartphone for a certain period of time	2.22(0.82)	−.056	.141	.575	.228
19	I feel the urge to use my smartphone again right after I stopped using it	2.15(0.80)	.097	.372	.404	.068
3	I find that I have been hooking on smartphone longer and longer	2.38(0.75)	.032	−.086	.087	.867
9	I have increased substantial amount of time using smartphone per week in recent 3 months	2.15(0.80)	.092	−.002	−.106	.847
1	I was told more than once that I spent too much time on smartphone.	2.04(0.76)	.218	.124	.189	.302

### Internal consistency and test-retest reliability

The Cronbach's alpha for the total scale was 0.94, and for the four factors, “compulsive behavior”, “functional impairment”, “withdrawal”, and “tolerance” were 0.87, 0.88, 0.81, and 0.72, respectively. We also recruited 85 participants to examine a two-week test-retest reliability (Intra-class correlations) of the SPAI and its 4 subscales, resulting in 0.80–0.91 (*p*<0.001).

### Correlations between smartphone addiction and phantom vibration/ringing

Table 2 reveals that the four subscales of SPAI had moderate to high inter-factor correlations (0.56–0.78). The phantom vibration did not present significant correlation with any subscale of SPAI. The phantom ringing had very low correlation to “compulsive behavior” and “functional impairment”, but no association with “withdrawal” or “tolerance”.

**Table 2:** Correlations, means, and standard deviations for the subscales of Smartphone Addiction Inventory (SPAI) and phantom vibration/ringing syndrome.

	Compulsive behavior	Functional impairment	Withdrawal	Tolerance	Phantom vibration	Phantom ringing
Compulsive behavior	-					
Functional impairment	0.780**	-				
Withdrawal	0.674**	0.583**	-			
Tolerance	0.593**	0.569**	0.563**	-		
Phantom vibration	0.148	0.149	0.136	0.046	-	
Phantom ringing	0.173**	0.198**	0.120	0.129	0.397**	-
Mean (SD)	16.69(4.33)	14.77(4.11)	13.53(3.49)	6.57(1.85)	2.04(0.81)	1.46(0.77)

**p≤0.01.

## Discussion

We developed the SPAI on the basis of the CIAS and established its four-factor structure: compulsive behavior, functional impairment, withdrawal, and tolerance, by exploratory factor analysis. Our findings demonstrated that smartphone addiction has several aspects similar to those of the substance related and addictive disorder in DSM-5. These subscales showed good internal consistency and acceptable 2-week test-retest reliability. Smartphone has the advantages of Internet connectivity, portability and real-time communication. The symptoms of smartphone addiction may thus differ from those in Internet addiction [5] or “problematic cellular phone use” [10]. For example, the item 25 “I cannot have meals without smartphone use” modified from original item belonged to the factor “time management problems” in CIAS, was classified as a withdrawal symptoms in SPAI.

“Compulsive behavior” has been regarded as the core of addiction, and widely been measured on individuals with alcohol dependence [12] and Internet addiction [13]. The item 7, “Although using smartphone has brought negative effects on my interpersonal relationships, the amount of time spent on Internet remains unreduced”, with the highest factor loading in the compulsive behavior covers two symptoms most associated with decision-making problem in previous study of problematic cell phone use [10]. It demonstrated that compulsive smartphone use could not be stopped even when the addictive individuals aware of the negative consequence. “Compulsive behavior” in SPAI included the items of the four factors, tolerance, withdrawal, compulsion and interpersonal & health problems in the original CIAS. These items also covered the same items in “Daily-life disturbance”, “Positive anticipation”, “Withdrawal”, “Overuse”, “Tolerance”, but no item in “Cyberspace-oriented relationship” of Smartphone Addiction Scale (SAS) [6]. It implies not only the symptoms change from computer- to smartphone-related but also the potential for further classification in different samples.

The “functional impairment” includes (1) four of five identical items of functional impairment in Problematic Cellular Phone Use Questionnaire, (2) three items related to sleep problems derived from “time management problem” in CIAS and (3) item 24 involved in “increasing amount of time on smartphone” and “achieve same satisfaction as before”. The highlight of sleep related problems is consistent with the relationship between eveningness and compulsive Internet use in our previous research [13]. Epidemiological survey showed not just the Internet usage itself but also “screen time” affect sleep [14], and physiological study specified that blue light emitting diodes influence on the circadian system [15]. The evidence explained the same way in smartphone addiction. Two items, 12 and 24, had cross-loading in “functional impairment” and “compulsive behavior”. Since symptoms of smartphone addiction could cause the “functional impairment”, the cross-loadings existed.

Item 2, 4 and 16 of the six items in “withdrawal” derived from the same withdrawal items in CIAS. The item 2 and 4 also corresponded to the item 19 and 23 of the withdrawal factor in SAS. Besides, the item 25 is similar to the corresponding item “Bringing my smartphone to the toilet even when I am in a hurry to get there” in SAS. It described a unique withdrawal symptom of smartphone due to its portability. In item 14, the “eye opener” also presented in SAS, but it was emphasized the connection to social network. It is well known the patient with alcohol dependence is going through withdrawal in the morning, hence need for a drink as an “eye opener”[16]. Due to the portability of smartphone and the accessibility to Internet, the “eye opener” is an important and more frequent withdrawal symptom in smartphone addiction. The item 19 “feeling the urge to use my smartphone again right after I stopped using it” has cross-loading between “functional impairment” and “withdrawal”. In general, the withdrawal symptoms of substance did not occur “right after stopped it”. We preferred this item in “withdrawal” considering this special withdrawal symptom in smartphone use.

The factor “tolerance” has three items in SPAI but the factor loading is very high in the first two items. Tolerance was defined as spending more and more time on smartphone use, which was the same concept of tolerance in DSM [2] but different from the definition “always trying to control one's smartphone use but always failing to do so” in SAS [6]. However, it is very interesting that the tolerance factor has the lowest eigenvalue in both SPAI and SAS [6]. The different presentations of tolerance in smartphone from Internet addiction or substance use are noteworthy to be considered. Individuals have exchanged more and more information in their social network since the beginning of smartphone use. Like individuals with heavy use of cannabis who are generally not aware of having developed tolerance [17], the tolerance symptoms in smartphone addiction may be rarely identified. Tolerance may be difficult to determine by history taking alone when the substance used is mixed with other substances [17]. All participants in the study used smartphone and Internet in computer, for example, they can log in the social network by both ways. Thus, the tolerance should be reported by side information, such as item 1, i.e., “I was told more than once that I spent too much time on smartphone.” However, as the second prevalent symptoms in problematic cellular phone use in previous epidemiological survey, “tolerance” could differentiate those who had functional impairment caused by cellular phone use from those who had no functional impairment [10]. The evidence suggested tolerance is a meaningful symptom. The tolerance factor has the fewest (four) items in the original CIAS [5], and there were relative lack of the concept of “markedly diminished effect with continued use of the same amount” which is also an important aspect of tolerance in DSM [2]. In next revision, the concept should be added in.

We suggested the phantom vibration and ringing syndrome of smartphone are independent entities of smartphone addiction based on the very low correlation. Even in the six-factor structure in SAS, phantom ringing could not be classified in any factors.

Compared with previous study [6], there are three major strengths of this study. First, the participants were male predominant college student, which are the most high-risk group in substance and Internet addiction [7]. Second, the four-factor structure of SPAI is more consistent with the four components, i.e., excessive use, withdrawal, tolerance, and negative repercussions, that all of the variants of Internet addiction shared [18]. Third, we used the standard definitions of tolerance and withdrawal in DSM rather than simply summarized the description of all items within the same factor.

There are several methodological limitations that should be noted when interpreting our findings. First, all investigations were self-reported, and a more objective method is required to examine the concurrent validity. For example, an application recorded the frequency and duration of the real-time smartphone use [19], [20]. Second, the sample contained only college students, which limits the generalization of the findings. Future studies need to evaluate the psychometric properties of this instrument in general population samples. Third, there are only three items in the tolerance factor, which should be expanded to make the structure more stable. Finally, as one of the pilot studies in this field, the theoretical base of the present study was relatively insufficient.

In summary, the results from this study provide evidence that the SPAI is a valid and reliable self-administered screening tool to identify smartphone addiction. The consistent taxonomy with substance related and addictive disorder in DSM implies the property of “addiction” identical in smartphone addiction.
